# XFlow: An algorithm for extracting ion chromatograms

**DOI:** 10.1371/journal.pone.0227659

**Published:** 2020-10-22

**Authors:** Mathew Gutierrez, Rob Smith

**Affiliations:** Department of Computer Science, University of Montana, Missoula, MT, United States of America; Pacific Northwest National Laboratory, UNITED STATES

## Abstract

Mass spectrometry is a fundamental tool for modern proteomics. The increasing availability of mass spectrometry data paired with the increasing sensitivity and fidelity of the instruments necessitates new and more potent analytical methods. To that end, we have created and present XFlow, a feature detection algorithm for extracting ion chromatograms from MS1 LC-MS data. XFlow is a parameter-free procedurally agnostic feature detection algorithm that utilizes the latent properties of ion chromatograms to resolve them from the surrounding noise present in MS1 data. XFlow is designed to function on either profile or centroided data across different resolutions and instruments. This broad applicability lends XFlow strong utility as a one-size-fits-all method for MS1 analysis or target acquisition for MS2. XFlow is written in Java and packaged with JS-MS, an open-source mass spectrometry analysis toolkit.

## Introduction

Mass spectrometry is a popular approach for measuring the sample-bound content and quantity of a variety of classes of molecules across a broad range of applications including pharmaceuticals, forensics, biochemistry, and food science. All applications of mass spectrometry have a common problem: the instrument itself does not directly provide measurements of molecules nor their identities, but rather produces raw data that must be rendered human-interpretable through the application of data processing algorithms.

According to community perceptions, advancements in software have lagged behind the steady pace of instrumentation advancements [1]. Unlike other computational science fields (such as genomics) where several foundational computational problems are regarded as solved, most mass spectrometry users feel that significant problems in computational mass spectrometry remain unsolved [1] despite (in some cases) dozens of published algorithms designed to address them [2]. Beyond user sentiment, the experimental influence of algorithm selection suggests that the analysis and advancement of computational mass spectrometry algorithms is a valuable pursuit [3].

Mass spectrometry systems generate datasets that quantify counts of charged particles at specific mass-to-charge (m/z) values. In liquid chromatography-mass spectrometry (LC-MS) systems, these measurements are taken over the time (retention time or RT) required for the molecules to elute from a chromatography column designed to slow or speed the migration of the molecules depending on particular physico-chemical properties such as size, or polarity.

**Fig 1:**
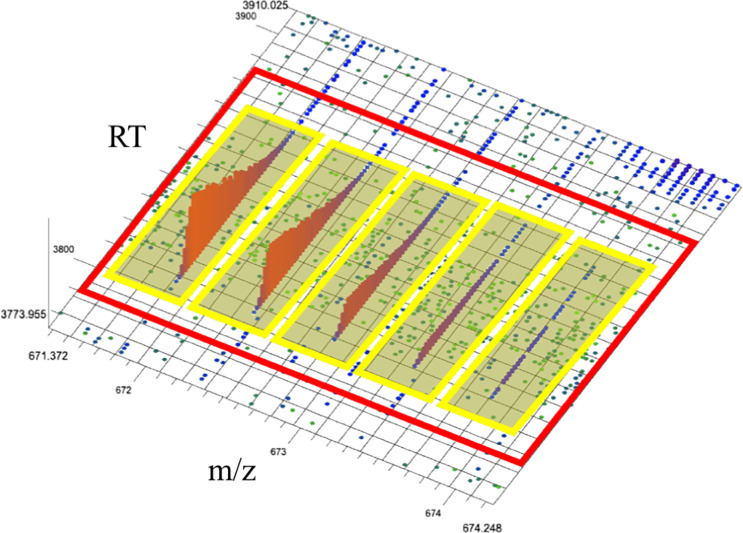
3d Isotopic envelope. In this figure are five extracted ion chromatograms (XIC) bounded by yellow rectangles. Each XIC is composed of points, each with m/z, RT and intensity (denoted by color and height on z axis). Each XIC is the evidence of an isotope of a molecule or molecules. The group of five XICs is referred to as an isotopic envelope, or feature, seen bounded by the red rectangle.

Mapping the raw LC-MS data points to particular classes of molecule (say, a particular peptide at a particular charge state) provides both an accurate count of the relative abundance of that molecule class (through integrating the intensities in those points) and discriminatory information about the identity of the molecule(s), as the charge state and uncharged mass can be derived through the m/z gap present between isotopic-specific sub-signals (extracted ion chromatograms or XICs) in the signal (see Fig 1).

Some existing algorithms attempt to resolve the features directly from the point data (e.g. OpenMS FFC [4]). Other algorithms split this process into two steps. First, two step algorithms cluster points into XICs, sometimes called isotopic traces (or features [5]) Second, by clustering XICs into isotopic envelopes (sometimes also called features [6]) (see Fig 1). This two-stage approach maximizes the utilization of available information and serves to reduce the amount of data by allowing a summary of each XIC to be used to find isotopic envelopes instead of cumbersome point data.

**Fig 2:**
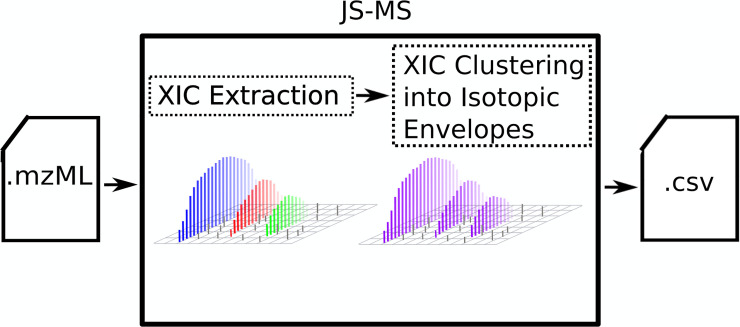
Ion chromatogram extraction in context. Like several other ion chromatogram extraction algorithms, XFlow forms one step in a two-step process where ion chromatograms (blue, red, green) are extracted from raw data. In the example workflow provided for the software JS-MS [8, 9], the XICs are then combined into isotopic envelopes (purple).

This manuscript presents *XFlow*, a novel algorithm for extracting ion chromatograms from LC-MS data. XFlow outperforms existing XIC algorithms evaluated recently on a benchmark human-curated dataset and provides qualitative evidence in support of high-function on alternative datasets. The output of XFlow can be used in conjunction with the XIC clustering algorithm Xnet [7] to map raw data points from an LC-MS run into the signal groups necessary for further analysis (see Fig 2).

A recent XIC benchmark study [10] noted that Massifquant [11], a Kalman filter-based XIC algorithm, performed well against other popular algorithms on a large set of hand-annotated XICs. Massifquant uses a Kalman filter to model XICs as time-series events where the probability of membership of a proximate point in the next scan is a factor of the m/z of previous points in the putative XIC. Massifquant has several drawbacks. It has a very large possible parameter space, takes considerable time to run, and lacks an objective or automatic approach to optimize parameters. Still, it outperformed all other evaluated algorithms on a large human-curated dataset.

Perhaps the principle theoretical advantage of Massifquant is that it attempts to assemble point membership in XICs as a function of the probability of a given point being a member of a given proto-XIC. Unfortunately, Massifquant uses Kalman filters—which require many expensive calculations), suffers from many parameters, and constructs XICs in decreasing time order which can lead to incorrect XICs.

Several algorithms have been published that avoid the theoretical and computational limitations of Massifquant by leveraging intensity order to iteratively construct XICs (for example, FFC [4], KPIC2 [5], and Agilent Profinder).

The core idea of intensity order XIC algorithms is the hypothesis that the most intense points in a mass spectrometry run also have the most accurate m/z measurement. Therefore, intensity of a point can be used as a surrogate for confidence. These algorithms incrementally adding points from surrounding windows to putative features seeded with a point of locally maximal intensity, earning the nickname of “waterfall” methods. Other algorithms use less rich sources of information. Shape filters (for example, matchedFilter [12] and centWave [13]) tend to degrade at lower intensities and are expensive to optimize for each signal in a run. Massifquant [11] and MaxQuant [14] both build XICs scan by scan, though in reverse order. Due to the Gaussian shape of XICs, this guarantees that the least confident information is the most relied upon in both of these algorithms, ensuring suboptimal performance.

Like other intensity order algorithms, XFlow avoids the drawbacks of relying on specific shapes or expensive models. Unlike other intensity order algorithms, XFlow takes the intensity-confidence hypothesis a step further. Instead of processing local windows through “walking” through time on each side of an XIC’s intensity apex or extracting an XIC in a region of interest around a high intensity local maxima, XFlow processes each point in decreasing order without simplifying heuristics and post-processing. In other words, for each point in the run, XFlow will either add it to an already existing putative feature, or create a new putative feature seeded with the current point, in decreasing order of intensity. In addition to achieving single-pass efficiency, XFlow’s strategy avoids the post-processing step necessary for local-neighborhood algorithms that can’t distinguish between overlapping features on their own. XFlow also avoids the drawback of reliance on user parameters, which, as we will show, is a significant concern for other algorithms due to the effect parameter selection can have.

## Methods

XFlow casts ion chromatogram extraction as a clustering problem, where points are clustered into XICs (see Fig 2).

### Baseline correction

XFlow uses a baseline filter for both: 1) determining the subset of points that will be clustered into XICs and 2) determining whether a point is allowed to seed a putative XIC. The justification for this thresholding is two part. The primary consideration of thresholding is to limit the admission of noise into the final output, while the secondary consideration is to reduce the computational burden to only the relevant subset of the data.

XFlow automatically calculates the baseline threshold from the data by calculating the average intensity of all points in the run, on the assumption that most points in the run will be very low intensity. All points below the baseline are excluded from XIC consideration, and only points with intensity at least twice the baseline are allowed to seed new putative XICs. The study of when and where to apply intensity thresholding is an active and ongoing topic of research in our lab due to the difficulty of avoiding bias, limiting noise inclusion, and maximizing signal inclusion [15].

### XIC construction

Let (**P**) be the set of all points (p_i_) in a run with intensity equal to or greater than baseline. Let **W** represent the set of points that are plausible members of the same XIC as a given p_i_ such that the intensity of the point is less than p_i_’s intensity. In theory, **W** could be the entire run. In practice, and in the implementation of XFlow provided, the m/z width of two times the automatically-calculated resolution estimate (explained below) and the RT width of two times the sampling rate are sufficient to generate good results.

For each p_i_ in decreasing intensity order down to twice the baseline, each point w_j_ in **W** is sorted by decreasing distance. Until the intensity of p_i_ is exhausted, each w_j_ is linked to p_i_ with the difference between p_i_ and w_j_ scaled by their distance, and this value is subtracted from p_i_’s intensity (see Eq 1).

$$intensity(p_{i,t_{2}})=intensity(p_{i,t_{1}})-\left|(intensity(p_{i,t_{2}})-intensity\left(w_{j}\right))*distance(p_{i},w_{j})\right|$$ (1)

Using intensity as a surrogate for likelihood of XIC membership, we estimate confidence of any link as a function of the difference of p_i_‘s intensity before and after linking the point divided by its intensity before linking (see Eq 2). A high confidence point is one that is near in space, and similar in intensity.

$$confidence=\frac{intensity(p_{i,t_{1}})-intensity(p_{i,t_{2}})}{intensity(p_{i,t_{1}})}$$ (2)

**Fig 3:**
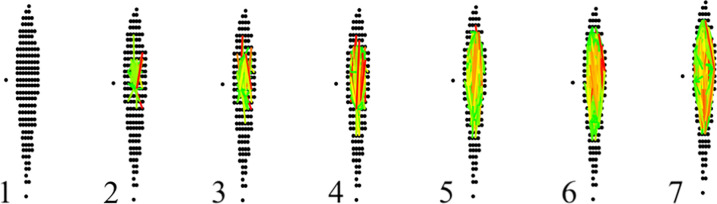
XFlow link progression. This figure details the linking process (lines) and associated confidence (color) of XFlow in a sequence of images with MZ and RT axis. Beginning with a completely unlinked region (1) XFlow links the highest intensity point to all nearby points (2). Linking continues in (3) and (4) as XFlow progressively considers lower and lower intensity points, creating links of varying confidence. Though this figure is just focusing on one XIC, the entire file is progressing in the same way simultaneously.

This value is stored such that each link in question has an associated confidence that is a function of the nearness in both intensity and Euclidean distance (given that the difference is scaled by their distance) (see Fig 3).

After links and confidence for those links are calculated for all (p_i_), XICs are extracted such that the points in each cluster consist of the highest confidence links when alternatives exist. Putative XICs are required to contain more than 5 points for centroided data and more than 15 points for profile data.

### No parameters

Unlike any published XIC algorithm, XFlow is also parameter-free. Unlike most XIC algorithms, XFlow is designed to be agnostic to instrument and to whether the data is centroided or profile. XFlow self-calibrates based on three parameters automatically derived from each run: resolution, sampling rate, and noise baseline. The baseline calculation is described above. The resolution is automatically estimated using the minimum m/z separation between any two points belonging to the same scan. The sampling rate is estimated by calculating the minimum RT separation between two consecutive scans.

### Evaluation

Algorithmic performance is evaluated on a hand-annotated dataset [16] from a recent study that presented over 57,000 XICs from a public LC-MS dataset [17] (UPS2). XFlow was compared to the algorithms centWave [13], matchedFilter [12], and MZMine2 [18], selected for comparison as equivalent open source algorithms. Accurate evaluation with respect to the hand annotated dataset required point by point comparison. For the chosen algorithms, point data was recovered using the window output that each provided.

For an XIC to be considered appropriately extracted, it must be matched to a corresponding hand annotated XIC. For the purposes of determining matches, we will refer to the set of points constituting an XIC produced by the software as **A** while the set of points constituting an XIC produced by hand annotation will be **H**. For an XIC to be considered correctly recovered, the sum of the intensity of the intersection of points between **A** and **H** must constitute greater than fifty percent of the sum of the intensity of the points in the hand annotated XIC (**H**). This fraction of shared intensity will be referred to as *S* (Eq 3).

$$S=\frac{\sum intensity(A⋂H)}{\sum intensity(H)}$$ (3)

## Results

**Fig 4:**
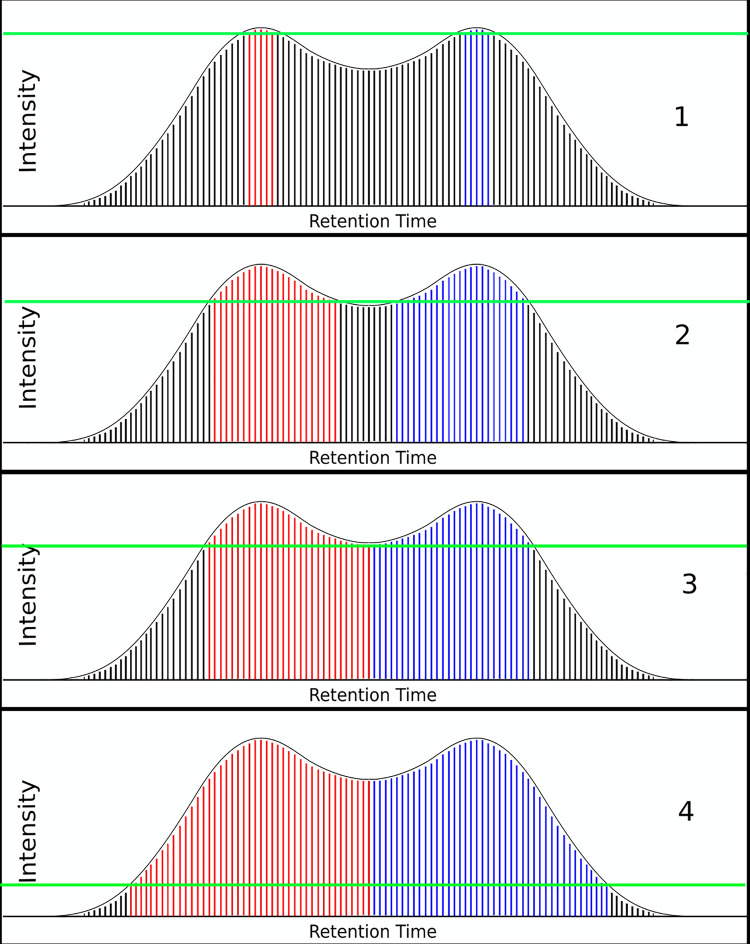
XFlow intrinsic deconvolution. This figure demonstrates the latent capacity for XFlow to deconvolve overlapping signals. Observe Subplot 1, XFlow has just begun linking points in intensity order, shown by the colored points. Next in subplot 2, XFlow has begin linking progressively less intense points to the higher intensity points. In subplot 3, XFlow links all points in the valley between two peaks, and all points are linked to one or the other growing XIC. In subplot 4, XFlow continues linking lower intensity peaks on either side of the bi-modal peak, adding points to each growing XIC respectively. Again note that while the figure is focusing on one XIC, in reality the entire file proceeds in this manner simultaneously.

Peak deconvolution, a problem generally thought of as difficult, is handled simply and intrinsically by XFlow’s intensity-first approach. This process is demonstrated in Fig 4 in a mockup of a bi-modal XIC with several steps between initial consideration and completion of linking.

**Fig 5:**
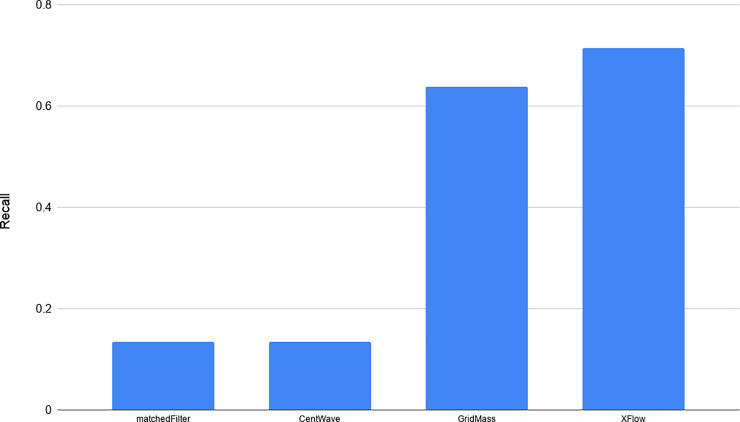
Percent recall of hand annotated XICs. Percentage of XICs shared between hand annotated dataset, and from the set of XICs output by each algorithm recovered from the UPS2 dataset. Note the disparity between algorithms with XFlow recovering the most with just over 70%, and matched filter recovering just over 15%.

We compared XFlow to several popular publicly available and functionally equivalent algorithms. For the purposes of this evaluation, algorithms were selected based on the similarity of the intended task (MaxQuant [14] and FFC [4], for example, do not stop at XIC extraction, but exports entire envelopes) and were limited according to previously published performance (KPIC2 [5], for example, was shown to perform comparably or worse than GridMass [18]). XCMS’s centWave [13] and matchedFilter [12] algorithms (optimized using Isotopologue Parameter Optimization [19]) and GridMass (optimized by employing grid search of parameters). Due to the difficulty of obtaining verified XIC datasets, quantitative validation of algorithmic results for XFlow, centWave, matchedFilter and GridMass are limited to the UPS2 dataset, the only dataset with hand annotated XICs. Five other reference or standard datasets were selected from the PRIDE repository: PXD000790, PXD000792, PXD003236, PXD008952, PXD011194. These additional files were selected in order to provide qualitative information. The RAW files were processed using ProteoWizard’s msConvert [20] (Version: 3.0.19277-b582d79cd) to create centroided and profile.mzml files using vendor centroiding algorithm for a total of 10 qualitative test files (5 profile, 5 centroided) The percent recall of each algorithm on the hand annotated dataset can be seen in Fig 5.

The hand-annotated dataset contains many accurate XICs, but is not a complete set of all XICs in the file. Without confidence in the existence of a complete set, there is no verifiable way to determine existence of false positives. Therefore, the best measure of false positives is given by Fig 5.

**Fig 6:**
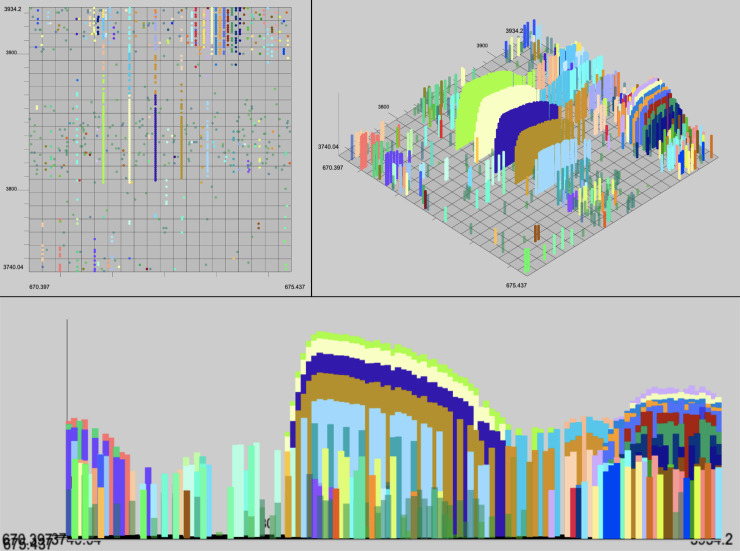
XFlow results. Selected result of XFlow on the hand annotated data in top-down, 3d and spectral views. All XICs in window are fully formed, and the extent of the XICs in the RT dimension are captured. Note the correctly distinguished bi-modal or tri-modal peaks in the spectral view.

**Fig 7:**
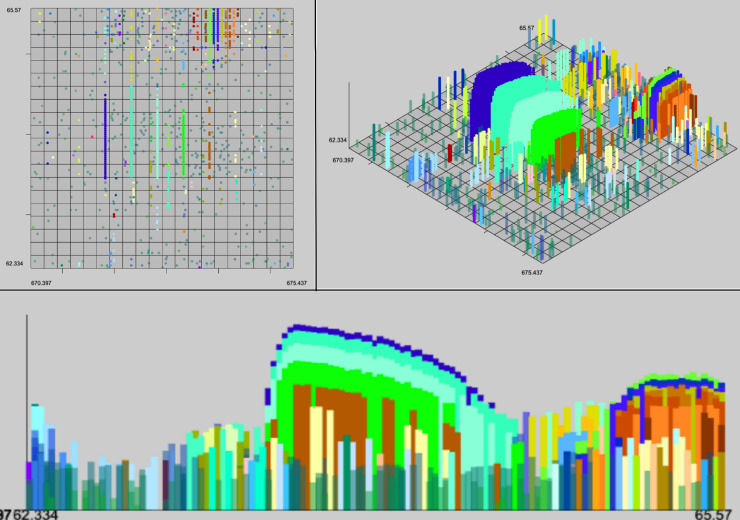
GridMass results. Selected result of GridMass on the hand annotated data in top-down 3d and spectral views. Most XICS in window are fully formed, and XICs extend in the RT dimension to their full extent. GridMass was also able to correctly distinguish bi-modal peaks after parameter optimization.

**Fig 8:**
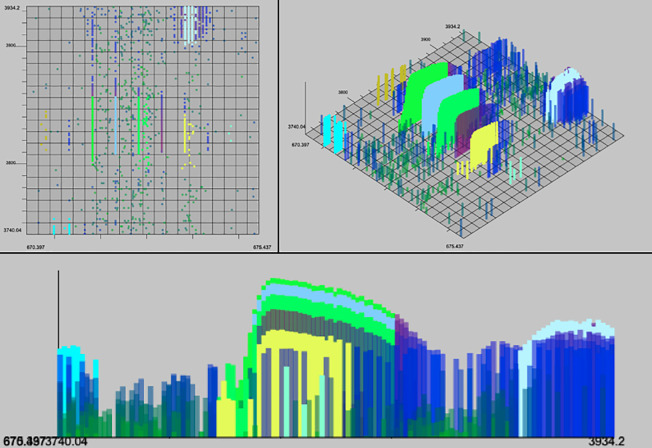
matchedFilter results. Selected result of matchedFilter on the hand-annotated data in top-down, 3d and spectral views. One XIC in the center has been completely skipped. Several XICs extend too far in the m/z dimension. XICs do not extend fully in the RT dimension.

**Fig 9:**
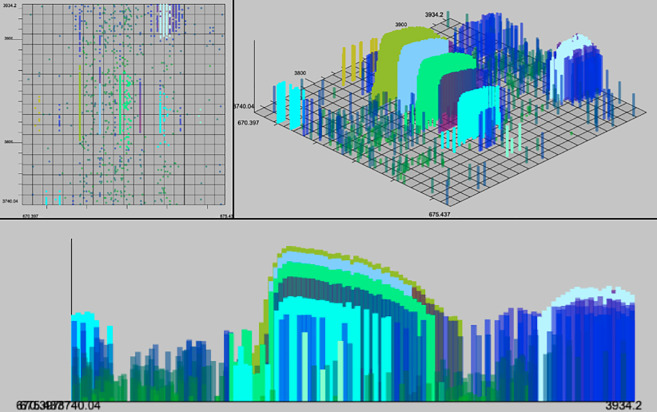
centWave results. Result example of centWave in top-down, 3d and spectral views. One XIC in the center has been completely skipped. Some XICs extend too far in the m/z dimension as before with matchedFilter. XICs also do not fully extend in RT dimension.

In addition to evaluating the performance of these algorithms quantitively we can analyze the output with a human eye. The characteristics of a high quality XIC are contiguity along retention time (RT), narrow span along the m/z axis, and a unimodal distribution of intensity along the RT axis. Example XICs from each algorithm on the UPS2 data are shown in Figs 6–9.

**Fig 10:**
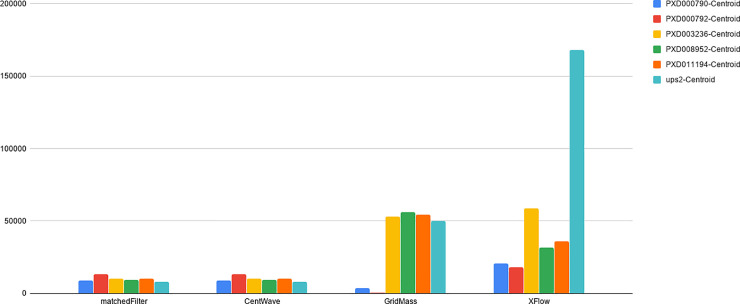
Total number of XICs for each centroid file. The number of XICs reported by each algorithm for each centroided dataset. Note the much larger set of XICs returned from XFlow for the UPS2 data set in comparison to other algorithms, and other files.

**Fig 11:**
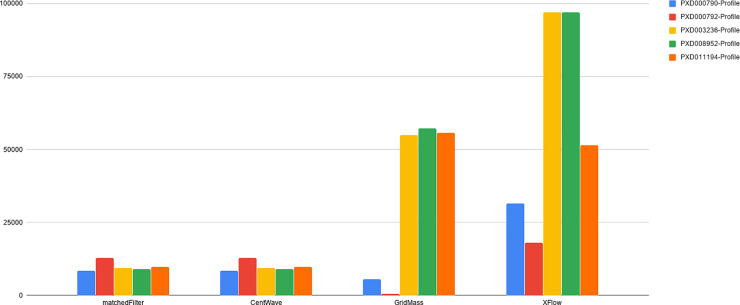
Total number of XICs for each profile file. The number of XICs reported by each algorithm for each profile dataset. Note some disparity among GridMass and XFlow between centroid (Fig 10) and profile datasets. Disparity between profile and centroid, while expected, is not desirable. Ideally, the centroid and profile datasets will have the exact same number of signals as they come from the same source. XCMS’s matchedFilter and centWave excelled at recovering the similar numbers of XICs from profile and centroided versions of the datasets. The effect of centroiding and quantifying the related loss of information during the process is an ongoing area of research in our lab.

The number of XICs recovered from alternative datasets for both centroid and profile data can be observed in Figs 10 and 11.

**Fig 12:**
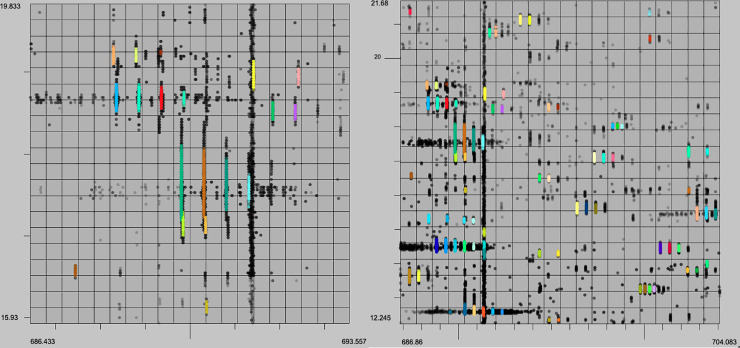
XFlow qualitative results. XFlow wide scale (left) and XFlow feature scale (right). It is clear that XFlow has self-adjusted the intensity slightly too high, and as a result is missing clearly present XICS (left). Further, XICs are well formed, and fully extended, having captured all relevant points.

**Fig 13:**
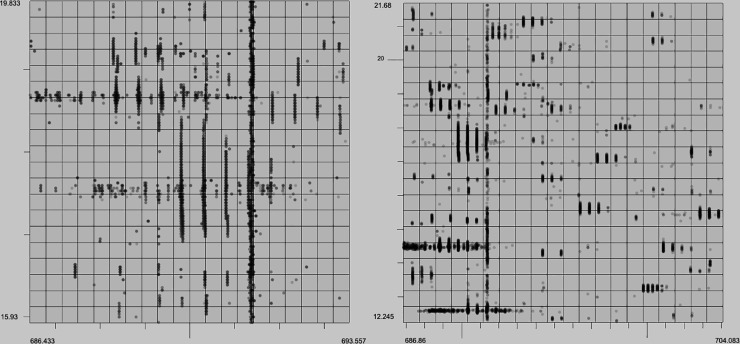
GridMass qualitative results. Feature view (left) and wide view (right). GridMass utterly failed to resolve any XICs in the selected window. It is clear that without a method to optimize the parameters, GridMass is unable to deliver appropriate results. So while approximately 50,000 XICs were recovered from the dataset, the qualitative quality of the XICs are poor.

**Fig 14:**
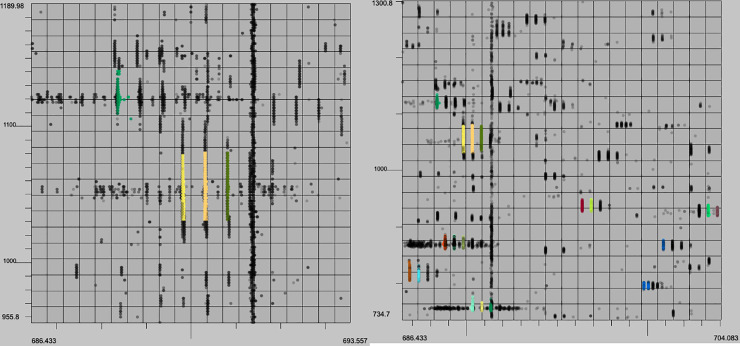
matchedFilter qualitative results. Feature view (left) and wide view (right) The left view shows that matchedFilter manages to return several of the XICs. MatchedFilter failed to find most of the high intensity points in the feature view (right), but found several of the peaks.

**Fig 15:**
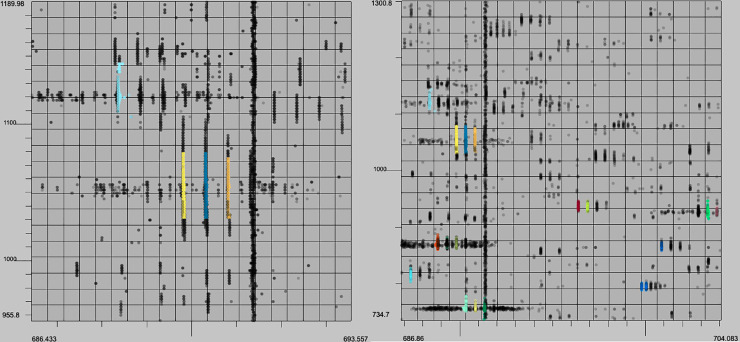
centWave qualitative results. Feature view (right) and centWave wide view (left) and the left view shows that centWave manages to return several of the XICs. Like matchedFilter, centWave failed to find most of the points in the XICs in the feature view (right), but found several peaks.

XCMS’ matchedFilter and centWave performed similarly in relation to each other. This is likely due to the common origin of the algorithms, and IPO’s optimization strategy. CentWave and matchedFilter also had the most similar results between centroid and profile data (Figs 9 and 10), also likely attributable to IPOs parameter optimization strategy. The downside of employing IPO is its very lengthy runtime. Further, while centWave and matchedFilter had a very high percentage of detected XICs matched to hand annotated XICs, the overall number of the hand annotated XICs they recovered is far fewer than XFlow or GridMass, qualitatively suggesting that they fail to recover lower intensity signals, an observation that can be verified by analyzing images from PXD011194 dataset to provide qualitative evaluation and comparison between algorithms tested. (See Figs 12–15).

**Fig 16:**
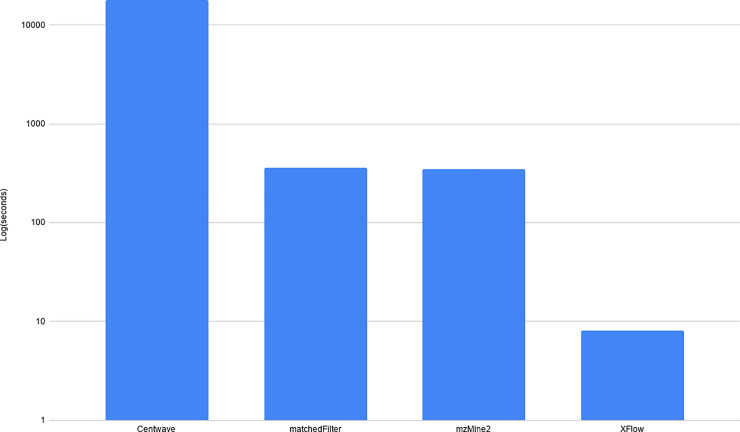
Runtime of algorithms on PXD000792. This chart shows the log scaled runtimes for each of the algorithms in seconds on the PXD000792-Centroid dataset. CentWave took the longest at ~18000 seconds (approximately 5 hours), matchedFilter and GridMass took nearly the same at about 5 minutes. XFlow took approximately 8 seconds. Note that the time consuming part of centWave and matchedFilter is the parameter optimization which necessitates repeated trials to obtain optimal results.

Additionally, it was observed that centWave and matchedFilter both harbor a tendency to over or under select around regions of interest (Figs 14 and 15). Additionally, with the prevalence of large datasets, the runtime of these algorithms is vitally important for their continued feasibility in the future. These runtimes can be seen in Fig 16 (note log scale).

**Fig 17:**
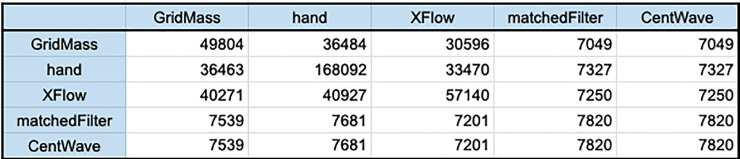
Comparison of Traces recovered for UPS2. This table was created to derive a metric to determine shared XICs recovered between algorithms and also to determine quality of deconvolution.

A comparison of the shared XICs found between the tested algorithms can be seen in *Fig 17*. Each algorithm is compared to every other algorithm and the hand annotated dataset twice to give some sense of the peformance of the algorithm. The directionality of the comparison is important, as each XIC present in the second comparison file can be matched to as many XICs in the first comparison file. This evaluation was conducted in this way to detect a failure of the algorithms to separate bimodal peaks. In this way, the difference between the two comparisons between the same algorithms can be determined to mean that the algorithm with more matches was better able to separate co-eluting XICs as it matched multiple XICs to one presumably concatenated bi-modal signal.

## Discussion

The results of this study have brought to light several interesting and key features of the ability of the evaluated algorithms to recover XICs from LC-MS data sets.

GridMass and XFlow were generally the most permissive algorithms tested, resulting in the most XICs recovered. Both algorithms also managed to recover many XICs from the UPS2 dataset, GridMass recovering just over 64%, and XFlow recovering 71%. XFlow recovered a disproportionally large number of XICs from the UPS2 dataset. Likely due to the large number of extremely low intensity noise points that drove the baseline calculation down. While GridMass performed very well on recovering the hand annotated data, it was optimized using a grid search evaluated using the hand annotated data, and was unable to replicate the success without this optimization strategy.

Considering the total number of XICs collected for each algorithm with respect to each data file, it is clear here that the UPS2 dataset has interesting qualities in relation to the other datasets (Fig 9). XFlow returned the most hand annotated XICs between any algorithm, this disparity seems only attributable to something inherent in the dataset itself, in addition to what was mentioned before, UPS2 has a relatively small difference between the signal intensity and the background noise intensity in the dataset.

The runtime of the algorithms is highly disparate, and the challenges of optimizing a highly parameterized algorithm such as centWave, matchedFilter, and GridMass (Even using an automated tool like IPO) is prohibitively time consuming for larger datasets. It is feasible to reuse optimized parameters but doing so is likely to return suboptimal results. In this way, it is clear that algorithms designed without user tunable parameters will excel.

## Conclusion

The size of the datasets, the complexity of the signals, and the noise obfuscation make XIC acquisition from MS1 data extremely challenging. The general method to account for complexity has been to include parameters to increase the scope of an individual algorithm. It was our goal in our lab to reduce complexity, and simplify the experience of conducting MS1 analysis by designing XFlow in a procedurally agnostic way such that it works on a wide variety of MS1 datasets without parameter modification regardless of centroiding or instrument type, a goal that is now accomplished. It’s clear that XFlow excels at signal acquisition for the UPS2 dataset in particular and performs favorably with respect to other algorithms in signal acquisition from alternate datasets (Figs 12–15). Additionally, while qualitative information is gained by comparing results for alternative datasets, it’s impossible to quantitatively evaluate the performance of the algorithms for datasets that do not have a hand annotated version. To this end, developing a database of a variety of hand annotated datasets with which to evaluate algorithms remains a valuable endeavor, in order to provide additional sources of comparison beyond UPS2. Further exploration into noise/signal detection and baseline detection would also serve to improve the predictability of XFlow in a method agnostic way.

## Supporting information

S1 File (TXT)
